# Evaluation of the MODS Culture Technique for the Diagnosis of Tuberculous Meningitis

**DOI:** 10.1371/journal.pone.0001173

**Published:** 2007-11-14

**Authors:** Maxine Caws, Dang Thi Minh Ha, Estee Torok, James Campbell, Do Dang Anh Thu, Tran Thi Hong Chau, Nguyen van Vinh Chau, Nguyen Tran Chinh, Jeremy Farrar

**Affiliations:** 1 Oxford University Clinical Research Unit, Hospital for Tropical Diseases, Ho Chi Minh City, Viet Nam; 2 Pham Ngoc Thach Hospital for Tuberculosis and Lung Diseases, Hung Vuong, Ho Chi Minh City, Viet Nam; 3 The Hospital for Tropical Diseases, Ho Chi Minh City, Viet Nam; 4 Centre for Clinical Vaccinology and Tropical Medicine, Churchill Hospital, Oxford, United Kingdom; University of Cape Town, South Africa

## Abstract

**Background:**

Tuberculous meningitis (TBM) is a devastating condition. The rapid instigation of appropraite chemotherapy is vital to reduce morbidity and mortality. However rapid diagnosis remains elusive; smear microscopy has extremely low sensitivity on cerebrospinal fluid (CSF) in most laboratories and PCR requires expertise with advanced infrastructure and has sensitivity of only around 60% under optimal conditions. Neither technique allows for the microbiological isolation of M. tuberculosis and subsequent drug susceptibility testing. We evaluated the recently developed microscopic observation drug susceptibility (MODS) assay format for speed and accuracy in diagnosing TBM.

**Methodology/Principal Findings:**

Two hundred and thirty consecutive CSF samples collected from 156 patients clinically suspected of TBM on presentation at a tertiary referal hospital in Vietnam were enrolled into the study over a five month period and tested by Ziehl-Neelsen (ZN) smear, MODS, Mycobacterial growth Indicator tube (MGIT) and Lowenstein-Jensen (LJ) culture. Sixty-one samples were from patients already on TB therapy for >1day and 19 samples were excluded due to untraceable patient records. One hundred and fifty samples from 137 newly presenting patients remained. Forty-two percent (n = 57/137) of patients were deemed to have TBM by clinical diagnostic and microbiological criteria (excluding MODS). Sensitivity by patient against clinical gold standard for ZN smear, MODS MGIT and LJ were 52.6%, 64.9%, 70.2% and 70.2%, respectively. Specificity of all microbiological techniques was 100%. Positive and negative predictive values for MODS were 100% and 78.7%, respectively for HIV infected patients and 100% and 82.1% for HIV negative patients. The median time to positive was 6 days (interquartile range 5–7), significantly faster than MGIT at 15.5 days (interquartile range 12–24), and LJ at 24 days (interquartile range 18–35 days) (P<0.01).

**Conclusions:**

We have shown MODS to be a sensitive, rapid technique for the diagnosis of TBM with high sensitivity, ease of performance and low cost (0.53 USD/sample).

## Introduction

Tuberculous meningitis (TBM) results in death or severe disability in as many as two thirds of patients [1]. The early diagnosis and instigation of chemotherapy is crucial to a successful outcome. In the absence of identification of drug resistance and institution of second line drug therapy, multi-drug resistant (MDR) TBM is always fatal [2]. However TBM diagnosis is difficult: the clinical presentation is diverse, rapid tests insensitive and the differential diagnosis is broad.

Three options are currently available for the rapid diagnosis of TBM: Ziehl-Neelsen (ZN) smear, molecular assays and diagnostic algorithms. In experienced hands, using large CSF volumes (>6 mls) and meticulous examination of slides (at least 30 minutes) the sensitivity of smear can exceed 60% [3], [4]. It is cheap and simple to perform. However, large volumes of CSF are rarely submitted to the laboratory, particularly from paediatric patients. Furthermore, in routine laboratories it is often not feasible to devote adequate time to examine a single specimen. Molecular techniques such as PCR assays require highly trained technicians, rigorous quality control to guard against contamination, are expensive and are not more sensitive than meticulous smear [5], [6]. Diagnostic algorithms based on simple clinical and laboratory features can be sensitive and specific [7] but require further evaluation, particularly in HIV-infected patients and children . ZN smear and diagnostic algorithms do not allow for the microbiological isolation of *Mycobacterium tuberculosis* (*M. tuberculosis*) or identification of drug resistance. By contrast, PCR can potentially combine identification and detection of drug resistance in one test.

Commercial rapid liquid culture techniques have greatly reduced turn around times for the isolation and drug susceptibility testing of mycobacteria [8]. They are, however, too expensive for routine use by most national tuberculosis control programs in developing nations and tend to be used on limited numbers of specimens in national reference laboratories.

Microscopic observation drug susceptibility assay (MODS) is a technique for the cheap, rapid identification of drug resistant *M. tuberculosis* through direct drug susceptibility testing (DST) in liquid culture [9]–[11]. Equipment requirements are minimal: a level 2 Biological Safety Cabinet and inverse light microscope. It has low cross-contamination rates and is suitable for use in high-burden settings [9].

It is unlikely that direct susceptibility testing from CSF specimens, as done with MODS on sputum specimens, will yield a high sensitivity due to the low numbers of bacilli in the primary specimen. Furthermore, in the comparative evaluation of diagnostic techniques for TBM, isolates are often seen to be positive by smear or PCR but negative by the more sensitive culture; this is thought to be due to the clumping of bacilli in aliquots of a sample [5]. These two factors are likely to lead to many uninterpretable and false positive results in direct susceptibility testing. We have therefore evaluated MODS as a technique for the rapid identification of *M. tuberculosis* in the CSF in comparison with MGIT and LJ culture against a clinical gold standard. Following primary isolation, samples can be inoculated for further DST, either by MODS, molecular techniques or conventional phenotypic DST, where available.

## Results

**Figure 1:**
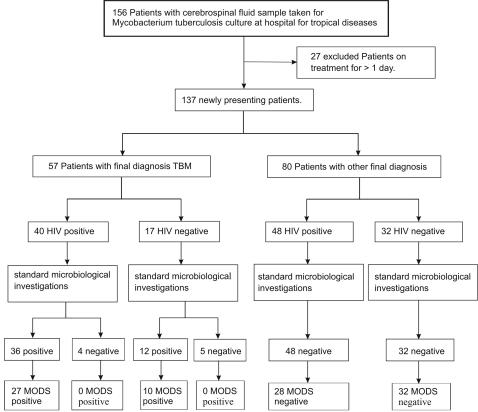
Flowchart of patients presenting to Hospital for Tropical Diseases with suspected TBM during the study showing results for standard microbiological investigations (MGIT/LJ/ZN smear) and MODS on CSF samples.

Two hundred and thirty samples were collected from 156 patients (figure 1). Sixty-one samples were from patients who were already on treatment (>1 day) for HIV associated TBM and were therefore analysed separately. Clinical data was not available for nineteen samples and these were therefore excluded from further analysis. One follow-up sample from a patient on treatment for 2 days had a contaminated MODS culture. This sample was positive by LJ and MGIT.

One hundred and fifty samples from 137 newly presenting patients remained. Fifty-seven (42%, n = 57/137) patients were deemed to have TBM by clinical diagnostic and microbiological criteria, excluding MODS (table 1). Of these patients, 30 were ZN smear positive, 27 of which were also positive by MODS. A further 27 were diagnosed with TBM clinically or by microbiological culture; ten of these were positive by MODS. For the nine patients without microbilogical confirmation of TBM, no other pathogen was isolated from the CSF, the CSF biochemistry was consistent with TBM and there was a response to TBM therapy or evidence of concurrent pulmonary TB.

**Table 1:** Features of 57 newly presenting patients with a final diagnosis of TBM.

patient number	HIV status	Final diagnosis	MODS (days)	ZN-smear	MGIT (days)	LJ (days)	CSF white cell count (cells/µl)	CSF protein (g/L)	CSF glucose (mmol/L)	CSF/blood glucose ratio	supplementary information
6	neg	TBM	Neg.	Neg.	Neg.	Neg.	200	1.9	3.3	0.36	PTB, smear neg
10	neg	TBM	+.(4)	+.	+(10)	+ (14)	1450	1.2	0.8	0.5	miliary PTB
12	+	TBM	Neg.	Neg.	Neg.	+ (65)	34	1.7	3	0.55	
13	+	TBM	Neg.	Neg.	Neg.	Neg.	1		<1		
14	+	TBM	+.(6)	+.	+(17)	+(17)	275	1.1	3.4	0.63	
15	+	TBM	+.(6)	+.	Neg.	+(13)	110	1.8	5.5	0.18	PTB, died
16	+	TBM	+.(3)	+.	Neg.	+(13)	160	2.4	0.5	0.10	PTB
18	neg	TBM	+.(14)	neg	Neg.	+(57)	270	1.6	0.5	0.10	
23	+	TBM	+.(7)	+.	+(18)	+(18)	116	1.4	1.9	0.31	PTB, dilated ventricles on CT
30	+	TBM	+.(6)	+.	+(50)	Neg.	62	1.3	2.2	0.43	
31	+	TBM	Neg.	Neg.	Neg.	Neg.		0.6	1.5	0.38	PTB
33	+	TBM	Neg.	Neg.	Neg.	Neg.	60	1.6	1.7	0.33	
36	+	TBM	Neg.	Neg.	Neg.	Neg.		1.6	1.5	0.39	
38	+	TBM	+.(6)	+.	+(35)	+(20)	695	1.4	2.7	0.38	
40	neg	TBM	+.(4)	+.	+(32)	+(18)	750	1.3	2	0.36	
43	+	TBM	+.(21)	Neg.	+(29)	Neg.	102	1.5	1.8	0.30	
44	neg	TBM	+.(7)	Neg.	+(29)	+(37)	650	1.34	1.6	0.2	PTB
46	neg	TBM	+.(10)	Neg.	+(17)	+(40)	480		<1		miliary TB . Abnormal CT
52	neg	TBM	+.(6)	Neg.	+(14)	+(31)	60	1.7	<0.5		
53	neg	TBM	Neg.	Neg.	Neg.	Neg.	224	2.1	2.1	0.40	
55	+	TBM	+.(7)	+.	+(14)	+ (14)	675	1.6	2	0.5	miliary TB
66	+	TBM	Neg.	+.	Neg.	+(25)	40	2.2	1.3	0.22	miliary TB, died
68	+	TBM	+.(7)	Neg.	+(16)	+(30)	685	0.95	2.5	0.33	
78	+	TBM	+.(4)	+.	+(8)	+(14)	2980	2.4	1.3	0.30	CT consistent with TBM, PTB
88	neg	TBM	Neg.	Neg.	+(21)	+(55)		1.5	2	0.40	
89	+	TBM	+.(30)	Neg.	+(21)	+(55)	510	1.7	1.6	0.29	
92	+	TBM	Neg.	+.	Neg.	+(70)		2.3	1.2	0.23	
96	+	TBM	Neg.	Neg.	+(31)	+(55)	293	1.2	2.4	0.49	CT consistent with TBM,
105	+	TBM	+.(4)	+.	+(35)	+(35)	350	1.6	<0.5		
106	+	TBM	+.(6)	+.	+(17)	+(21)	218	2.1	<0.5		
127	+	TBM	Neg.	Neg.	+(32)	+(32)	2010	1.2	2.2	0.42	CT consistent with TBM,
129	neg	TBM	+.(7)	+.	Neg.	Neg.		1.5			
131	+	TBM	+.(8)	Neg.	+(13)	+(21)	590	1.4	1.6	0.42	miliary TB
136	+	TBM	+.(6)	+.	+(13)	+(17)	375	1.2	<0.5		
138	+	TBM	+.(5)	+.	+(8)	+(26)	180	1.3	1.4	0.18	CT consistent with TBM, PTB
140	+	TBM	Neg.	Neg.	+(28)	+(63)	10	0.6	2.3	0.38	fungi neg
141	neg	TBM	Neg.	Neg.	Neg.	Neg.	210	1	2.8	0.64	
143	+	TBM	+.(4)	+.	+(7)	+(28)	3950	1.4	1.3	0.33	CT consistent with TBM
144	neg	TBM	Neg.	Neg.	Neg.	Neg.		0.8	1.6	0.30	
146	+	TBM	+.(4)	Neg.	+(15)	+(28)	149	0.32	1.6	0.22	
148	+	TBM	+.(7)	+.	+(14)	+(21)	1475	1.2	<0.5		
152	neg	TBM	+.(6)	Neg.	+(14)	+(21)	238	0.8	<0.5		
157	+	TBM	+.(7)	+.	+(11)	+(18)	1290	0.5	2.5	0.36	CT consistent with TBM, PTB, completed treatment of tuberculous lymphadenitis previously
159	neg	TBM	Neg.	Neg.	Neg.	Neg.		1.2	2.8	0.68	
166	+	TBM	Neg.	+.	Neg.	Neg.	140	0.5	2.5	0.36	CT consistent with TBM, PTB
167	+	TBM	+.(6)	+.	+(4)	+(17)	340	1.3	2	0.45	CT consistent with TBM
176	+	TBM	+.(6)	+.	+(13)	+(16)	142	1.5	2	0.33	
179	+	TBM	Neg.	Neg.	+(13)	Neg.	505	1.2	1.8	0.63	
181	+	TBM	Neg.	Neg.	+(12)	+(27)	284	1.3	3	0.6	lymph node AFB smear (+)
182	+	TBM	+.(5)	+.	+(12)	+(22)		1.5	<0.5		
194	+	TBM	+.(5)	+.	+(13)	Neg.	2460	1.6	1.3	0.17	Encephalitis on CT
197	+	TBM	+.(7)	+.	+(34)	Neg.	233	1.7	1.5	0.34	
200	neg	TBM	+.(6)	+.	+(15)	+(18)	410	not done	1.4	0.22	CT consistent with TBM, PTB
201	neg	TBM	Neg.	Neg.	+(12)	+(15)		1.2	2.1	0.28	
204	neg	TBM	+.(8)	+.	+(11)	+(30)	1065	1.2	3.2	0.51	
207	+	TBM	+.(7)	+.	+(24)	+(24)	544	1.6	2.1	0.29	pneumonia on CXR
211	+	TBM	+(5)	+.	+(22)	Neg.	644	0.9	3.1	0.62	

CXR = chest X-ray, CT = computerised tomography of the brain, TBM = tuberculous meningitis, + = positive, neg = negative, AFB = Acid fast bacilli, PTB = pulmonary tuberculosis.

Eighty patients did not fulfil the diagnostic criteria for TBM and were negative by ZN smear, MODS, LJ, MGIT. Eighty-eight patients (n = 88/137, 64.2%) were HIV-infected. A summary of the patient data is presented in tables 1 (TBM patients) and 2 (other diagnoses).

When analysed by patient, sensitivity of MODS was 90.0% [95% C.I 79.2–100] (n = 27/30), 80.4% [95% C.I. 68.4–91.6] (n = 37/46) and 64.9% [95% C.I 52.7–77.3] (n = 37/57) against smear, culture and clinical criteria respectively (table 3). With a clinical gold standard the sensitivity of smear was 53% [95% C.I. 40.1–65.9] (n = 30/57) and of both culture techniques, LJ or MGIT 70.2% [95% C.I. 58.0–82.0] (n = 40/57). There was 91.9% agreement between the MODS and MGIT tests, Kappa = 0.801. For MODS and LJ, agreement was 89.0%, kappa = 0.728; for MGIT and LJ there was 91.2% agreement, kappa = 0.788. Sensitivity and specificity data is summarised in table 4.

**Table 2:** Features of 80 newly presenting patients with a final diagnosis other than TBM.

patient number	HIV status	Final diagnosis	MODS (days)	smear	MGIT (days)	LJ (days)	CSF white cell count	CSF protein (g/L)	CSF glucose (mMol/L)	CSF/blood glucose ratio	supplementary information
1	+	Cryptococcal meningitis	Neg.	Crypto	Neg.	Neg.	55	0.5	2.2	0.55	Abnormal CXR, died, Cr Ag CSF (+)
2	neg	Herpes simplex encephalitis	Neg.	Neg.	Neg.	Neg.	115	1.7	3.2	0.58	CSF PCR HSV (+)
3	neg	Bacterial menigitis	Neg.	cocci	Neg.	Neg.	820	1	1.92	0.53	blood culture: S..suis (+)
5	+	Cryptococcal meningitis	Neg.	Crypto	Neg.	Neg.	253				treatment of Latent TB for 3 months.
7	+	Cryptococcal meningitis	Neg.	Crypto	Neg.	Neg.	6	1.7	2.5	0.45	Cr Ag CSF (+)
8	neg	Bacterial menigitis	Neg.	gram +	Neg.	Neg.		2	<0.5		
19	+	Cryptococcal meningitis	Neg.	Crypto	Neg.	Neg.	45	0.7	0.8	0.13	died
21	neg	Cryptococcal meningitis	Neg.	Neg.	Neg.	Neg.	399		1.5	0.27	Cr Ag serum (+)
24	neg	Viral Encephalitis	Neg.	Neg.	Neg.	Neg.		1.3	2.3	0.55	
32	+	Cryptococcal meningitis	Neg.	Crypto	Neg.	Neg.			2.6	0.52	
34	neg	eosinophillic meningitis	Neg.	Neg.	Neg.	Neg.	260	1.1	2.2	0.39	normal CXR
37	neg	meningoencephalitis	Neg.	Neg.	Neg.	Neg.	131		2.7	0.52	
42	+	Cryptococcal meningitis	Neg.	Neg.	Neg.	Neg.	64	2	3.4	0.34	Cr Ag CSF : (+)
47	+	Cryptococcal meningitis	Neg.	Crypto	Neg.	Neg.	180		1.2	0.19	
49	+	Cryptococcal meningitis	Neg.	Crypto	Neg.	Neg.	50	0.4	3.5	0.53	died
50	+	Bacterial meningitis	Neg.	Neg.	Neg.	Neg.			low		
54	+	Cryptococcak meningitis	Neg.	Crypto	Neg.	Neg.	21		3.1	0.62	Cr Ag CSF (+), normal CXR
57	neg	Viral Encephalitis	Neg.	Neg.	Neg.	Neg.	44	0.8	3.4	0.50	
61	neg	Viral Encephalitis	Neg.	Neg.	Neg.	Neg.	89	0.8	1.9	0.35	
62	+	Cryptococcall meningitis	Neg.	crypto	Neg.	Neg.	147	1.9	3.2	0.58	pneumonia on CXR, normal CT
64	+	Cryptococcal meningitis	Neg.	Neg.	Neg.	Neg.	47	0.7	2.7	0.42	Cr Ag CSF : (+)
67	+	Cryptococcal meningitis	Neg.	Crypto	Neg.	Neg.					
72	+	Toxoplasmo encephalitis	Neg.	Neg.	Neg.	Neg.	16	2.1	3	0.57	CSF: crypto (−)
73	+	Cryptococcal meningitis	Neg.	Crypto	Neg.	Neg.	2		2.2	0.35	
76	+	Bacterial meningitis	Neg.	Neg.	Neg.	Neg.	4	1.5	3.2	0.53	CSF: crypto (−)
79	+	Toxoplasma encephalitis	Neg.	Neg.	Neg.	Neg.		1.7	1.7		CSF: crypto (−)
81	+	Cryptococal meningitis	Neg.	Crypto	Neg.	Neg.	16		<0.5		
83	+	Cryptococcal meningitis	Neg.	Crypto	Neg.	Neg.			<0.5		
84	neg	Bacterial meningitis	Neg.	Neg.	Neg.	Neg.	531		2.7	0.43	
90	+	Fungal meningitis	Neg.	Neg.	Neg.	Neg.			1.3	0.21	
91	+	Fungal meningitis	Neg.	Neg.	Neg.	Neg.	38	1.4	2.2	0.40	
97	neg	Viral encephalitis	Neg.	Neg.	Neg.	Neg.		1.3			
98	+	Crypococcus meningitis	Neg.	Neg.	Neg.	Neg.	7		2.8	0.61	Cr Ag (+)
99	neg	Viral encephalitis	Neg.	Neg.	Neg.	Neg.	920	1.9	1.5	0.21	normal CXR, normal CT
102	neg	Bacterial meningitis	Neg.	Neg.	Neg.	Neg.	4	0.7	2.5	0.63	
108	+	Cryptococcal meningitis	Neg.	Neg.	Neg.	Neg.	210	1.5	1.3	0.27	Cr Ag CSF (+)
111	+	Bacterial meningitis	Neg.	Neg.	Neg.	Neg.	1735	0.9	3.1	0.51	
112	+	Fungal meningitis	Neg.	fungus	Neg.	Neg.	20	2	<0.5		
114	+	meningoencephalitis	Neg.	Neg.	Neg.	Neg.	4	1.2	1.7	0.24	pneumonia
116	+	Cryptococal meningitis	Neg.	Neg.	Neg.	Neg.	327	1.8	2.1	0.36	Cr Ag CSF (+)
117	neg	Bacterial meningitis	Neg.	cocci	Neg.	Neg.	2500	1.7	<0.5		alpha haemolytic streptococcus species.
118	neg	Bacterial meningitis	Neg.	Neg.	Neg.	Neg.	600	1.8	1.21	0.14	pneumonia on CXR, BAL: Staph.aureus, Pseudomonas aeruginosa
119	neg	Eosinophillic meningitis	Neg.	Neg.	Neg.	Neg.		49			BAL: pseudomonas.
125	+	meningitis	Neg.	fungus	Neg.	Neg.	60	0.6	<0.5		
128	+	Cryptococcus meningitis	Neg.	crypto	Neg.	Neg.	11	0.9	2.6	0.48	Cr Ag CSF (+)
130	+	Fungal meningitis	Neg.	Neg.	Neg.	Neg.		34			
132	+	Fungal meningitis	Neg.	fungi	Neg.	Neg.	4	1	1.7	0.30	
134	+	Fungal meningitis	Neg.	Neg.	Neg.	Neg.	4	1	2.3	0.53	
139	neg	Eosinophillic meningitis	Neg.	Neg.	Neg.	Neg.	620	0.8	3.0	0.50	Abnormal CT, normal CXR, Serodiagnosis: cysticencus cellulosae
142	neg	Bacterial meningitis	Neg.	Neg.	Neg.	Neg.	900	0.7	1.7	0.16	normal CXR
145	+	Cryptococcal meningitis	Neg.	Neg.	Neg.	Neg.	33	0.68	2.9	0.58	CSF Culture: crypto (+)
155	+	Toxoplama	Neg.	Neg.	Neg.	Neg.	4	0.6	3.6	0.59	
158	+	Cryptococcall meningitis	Neg.	crypto	Neg.	Neg.	11	0.6	2.5	0.46	Cr Ag (+),CSF culture (+) with crypto, normal CXR
160	+	Cryptococcal meningitis	Neg.	crypto	Neg.	Neg.	140	1.3	0.6	0.11	CSF culture: Crypto (+), normal CXR
161	neg	bacterial meningitis	Neg.	Neg.	Neg.	Neg.	32	0.5	2.7	0.30	normal CT
162	neg	Viral meningitis	Neg.	Neg.	Neg.	Neg.	70	1.1	2.1	0.49	odema on CT
163	neg	Bacterial meningitis	Neg.	Neg.	Neg.	Neg.	80	0.6	3.1	0.70	
164	neg	Bacterial meningitis	Neg.	Neg.	Neg.	Neg.	384	0.8	2.6	0.57	
171	+	Fungal meningitis	Neg.	fungus	Neg.	Neg.	9	not done yet	1.4	0.4	recieved fungal treatment 12 days previously, normal CXR.
172	+	Bacterial meningitis	Neg.	Neg.	Neg.	Neg.		0.7	3.1	0.6	
173	neg	Viral meningitis	Neg.	Neg.	Neg.	Neg.	370	1	2.8	0.46	
175	neg	Meningoencephalitis	Neg.	Neg.	Neg.	Neg.	1	0.3	3.1	0.6	normal CT
184	+	Meningitis	Neg.	Neg.	Neg.	Neg.	4	0.8	3.4	0.83	
185	neg	Viral Encephalitis	Neg.	Neg.	Neg.	Neg.		0.8	4.1	0.68	
188	+	Fungal meningitis	Neg.	fungal	Neg.	Neg.	1	0.6	2.3	0.49	Abnormal CT, lesion on CXR,
189	+	Fungal meningitis	Neg.	fungal	Neg.	Neg.			1.3		
190	+	Fungal meningitis	Neg.	fungal	Neg.	Neg.	1	0.6	3.2	0.60	
192	+	Fungal meningitis	Neg.	fungal	Neg.	Neg.	18	0.8	3.2	0.59	
206	neg	Meningitis	Neg.	Neg.	Neg.	Neg.	22	0.2	4	0.57	
208	+	Fungal meningitis	Neg.	fungus	Neg.	Neg.	169	1.5	2	0.38	
209	+	Alcohol poisoning	Neg.	Neg.	Neg.	Neg.	153	1.2	2.3	0.50	normal CXR
215	neg	Meningitis	Neg.	Neg.	Neg.	Neg.	22	1.5	1.1	0.17	pneumonia
216	neg	Meningitis	Neg.	Neg.	Neg.	Neg.	965	0.4	4	0.48	
219	+	Bacterial meningitis	Neg.	Neg.	Neg.	Neg.	735	1	3.8	0.36	
220	neg	Bacterial meningitis	Neg.	Neg.	Neg.	Neg.	8500	0.9	5.3	0.55	
222	+	Meningitis	Neg.	Neg.	Neg.	Neg.		1.2			
223	+	Meningoencephalitis	Neg.	Neg.	Neg.	Neg.		0.8			
224	neg	Viral meningitis	Neg.	Neg.	Neg.	Neg.	142	0.7	3.5	0.70	normal CT
227	neg	Herpes simplex encephalitis	Neg.	Neg.	Neg.	Neg.	220	0.9	2.7	0.48	PCR HSV (+), abnormal CT, abnormal CXR
229	neg	Viral Encephalitis	Neg.	Neg.	Neg.	Neg.	102	0.9	3.2	0.80	normal CT, normal CXR

HSV = herpes simplex virus, CXR = Chest X-ray, CT = computerised tomography, Cr Ag = cryptococcus neoformans antigen, (+) = positive, Neg = negative.

**Table 3:** Results of MODS for 137 patients presenting with suspected TBM by final diagnosis.

clinical diagnosis	TBM	Non-TBM	Total
MODS positive	37	0	37
MODS negative	20	80	100
Total	57	80	137

**Table 4:** Sensitivity of all techniques analysed by sample (n = 63), patient (n = 57) and HIV status. Specificity of all techniques in all cases was 100%.

	Technique [% sensitivity (no. positive samples)]			
Group	smear	MODS	MGIT	LJ
Analysis by sample (n = 63)	52.4% (33)	65.1% (41)	73% (43)	68.3% (43)
Analysis by patient (n = 57)	53.0% (30)	64.9% (37)	70.2% (40)	70.2% (40)
HIV negative patients only (n = 17)	29.4% (5)	58.8% (10)	58.8% (10)	64.7% (11)
HIV positive patients only (n = 40)	62.5% (25)	67.5% (27)	75% (30)	72.5% (29)

Specificity of all techniques was 100% (n = 80/80). The sensitivity and specificity of MODS was 58.8% [95% C.I. 35.7–82.3] (n = 10/17) and 100% (n = 32/32) in HIV-uninfected patients and 67.5% [95% C.I. 53.5–82.5] (n = 27/40) and 100% (n = 48/48), in HIV-infected patients. The positive and negative predictive values were 100% and 78.7% in HIV-infected patients and 100% and 80.0% in HIV negative patients, respectively.

When analysed by sample against a clinical gold standard, the sensitivity and specificity of MODS for newly presenting patients was 65.1% [95% C.I. 53.2–76.8] (n = 41/63), and 100% (n = 87/87) respectively. The sensitivites by sample for smear, LJ and MGIT were 52.4% [95% C.I. 39.7–64.3] (n = 33/63), 68.3% [95% C.I. 56.4–79.6] (n = 43/63) and 73.0% [95% C.I. 62.0–84.0] (n = 46/63), respectively. The specificity of all methods was 100%. In 96 samples from HIV-infected patients, the sensitivity was 68.9% [95% C.I. 55.5–82.5] (n = 31/45) and specificity 100% (n = 51/51). In 54 samples from HIV-uninfected patients the sensitivity was 55.6% [95% C.I. 42.7–69.3] (n = 10/18) and specificity was 100% (n = 36/36).

**Figure 2:**
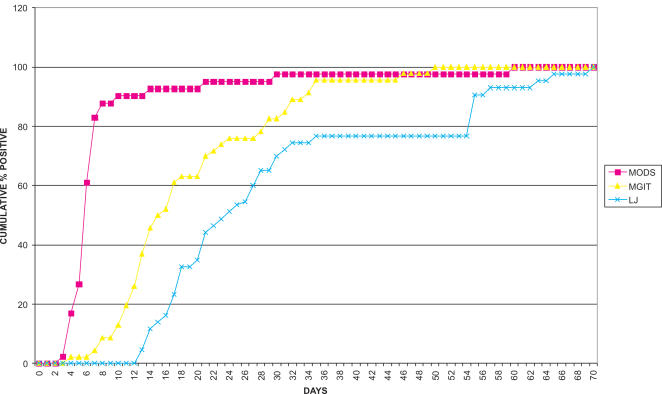
Cumulative percentage of positive cultures by day for MODS, MGIT and LJ culture.

The median time to detection of MODS positive cultures (n = 41) from newly presenting patients was 6 days (interquartile range 5–7 days). Ninety percent of samples (n = 37/41) were positive in ≤10 days. The median time to positive for MGIT (n = 46) and LJ cultures (n = 43) was 15.5 days (interquartile range 12–24 days) and 24 (interquartile range 18–35), respectively. MODS was significantly faster than MGIT and LJ (P<0.01), (Figure 2).

The mean CSF sample volume was 4.6 mls (range 1–14 mls). For MODS positive samples the average volume was 5.2 mls (range1–14 mls) and for MODS negative samples 4.4 mls (range 1–13 mls).

Of 61 CSF samples from 27 patients on TBM therapy for between 2 days and 9 months, 16 were positive by one of the four methods. Seven samples were positive by MODS. Eight samples were positive by smear, 10 by MGIT and 9 by LJ (table 5).

**Table 5:** Summary of 61 follow-up samples from 27 HIV-positive patients with HIV associated TBM on >7 days TB therapy*.

patient	treatment duration at sample date (days)	CSF volume (mls)	MODS (DAYS)	smear	MGIT (DAYS)	LJ (DAYS)	supplementary information
101	84	4	NEG	NEG	NEG	NEG	
113	210	2	NEG	NEG	NEG	NEG	
12	7	3	NEG	NEG	NEG	NEG	
12	28	3	NEG	NEG	NEG	NEG	
12	49	3	NEG	NEG	NEG	NEG	
12	84	2	NEG	NEG	NEG	NEG	
123	6	6	NEG	+	NEG	NEG	DIED
127	5	4	NEG	NEG	NEG	+ (28)	
127	28	3	NEG	NEG	NEG	NEG	
131	6	4	NEG	NEG	+ (14)	+ (21)	
131	28	1	NEG	NEG	+(15)	NEG	
143	2	2	cx	+	+ (13)	+ (23)	
15	6	5	NEG	NEG	NEG	NEG	DIED
150	2	9	+ (10)	+	+ (14)	+ (21)	
150	5	2	NEG	NEG	NEG	NEG	
150	28	2	+(7)	+	NEG	NEG	
165	42	3	NEG	NEG	NEG	NEG	
165	56	1	NEG	NEG	NEG	NEG	
166	3	4	NEG	+	NEG	NEG	
166	7	3	NEG	NEG	NEG	NEG	
166	28	2	NEG	NEG	NEG	NEG	
167	7	2	NEG	NEG	NEG	NEG	
167	28	1	NEG	NEG	NEG	NEG	
17	28	2	NEG	NEG	NEG	NEG	
194	2	3	NEG	+	NEG	NEG	
194	6	3	NEG	NEG	NEG	NEG	
202	84	3	NEG	NEG	NEG	NEG	
207	2	9	+ (7)	+	+(20)	+(20)	
207	7	7	NEG	NEG	NEG	NEG	
211	7	2	NEG	NEG	NEG	NEG	
23	2	4	NEG	NEG	NEG	NEG	
23	28	3	NEG	NEG	NEG	NEG	
23	56	3	NEG	NEG	NEG	NEG	
25	70	1	NEG	NEG	NEG	NEG	
25	168	2	NEG	NEG	+(12)	NEG	
28	252	5	NEG	NEG	NEG	NEG	DIED
30	28	1	NEG	NEG	NEG	NEG	
30	42	2	NEG	NEG	NEG	NEG	
30	70	3	+ (8)	NEG	+(17)	+(21)	
35	28	5	NEG	NEG	NEG	NEG	
41	49	2	NEG	NEG	NEG	NEG	DIED
41	70	3	NEG	NEG	NEG	NEG	DIED
55	21	3	NEG	NEG	NEG	NEG	
55	70	7	NEG	NEG	NEG	NEG	
58	56	3	NEG	NEG	NEG	NEG	
58	84	5	NEG	NEG	NEG	NEG	
63	56	2	NEG	NEG	NEG	NEG	
66	6	3	NEG	NEG	NEG	NEG	DIED
66	28	4	+ (16)	NEG	+(19)	NEG	DIED
78	5	6	+(6)	+	+(16)	+(23)	
78	21	2	+ (10)	NEG	NEG	NEG	
78	70	5	NEG	NEG	NEG	NEG	
80	56	1.5	NEG	NEG	NEG	NEG	
80	56	5	NEG	NEG	NEG	NEG	
9	28	3	NEG	NEG	NEG	NEG	
96	7	3	NEG	NEG	NEG	NEG	
96	28	2	NEG	NEG	NEG	NEG	
96	56	4	NEG	NEG	NEG	NEG	
96	84	3	NEG	NEG	NEG	NEG	

* TB therapy = rifampicin, isoniazid, pyrazinamide, ethambutol for 3 months, followed by rifampicin and isoniazid for 6 months. All patients also received adjunctive dexamethsone therapy.

Among the 80 patients not diagnosed with TBM, 34 had a final diagnosis of fungal menigitis, 6 viral meningitis, 14 bacterial meningitis and 3 toxoplasmosis. The majority of diagnoses in the remaining cases remained uncertain (table 2).

### Costs

The cost per test of MODS for diagnosis was 0.53 USD per sample.

## Discussion

MODS is a sensitive, rapid technique for the diagnosis of TBM. Although MGIT showed a slightly higher sensitivity in this study, the difference was not significant (65% vs. 73%, P = 0.335) and MODS is significantly faster (median 6 days vs. 15.5 days, P<0.01). A smaller volume of deposit was innoculated into the MODS culture than the MGIT culture (100 µl versus 250 µl) and this is likely to account for the slightly lower sensitivity.

The median time to a positive MODS culture shown here is slightly faster than the median detection time of 7 days for isolation of *M. tuberculosis* from sputum samples in a large evaluation of MODS in Peru [11]. This shorter time to detection is surprising due to the paucibacillary nature of CSF samples and may be because the NALC-NAOH decontamination of sputa samples reduces viability of the TB bacilli- prior decontamination is not required for CSF samples. Moore *et al*. have previously shown that the bacillary load of a sample (measured by bacilli/100fields on smear) has negligible impact on time-to culture positivity by MODS [11]. It may be possible to reduce time to detection with the introduction of confirmatory biochemical or molecular tests for early confirmation of *M. tuberculosis*. Studies to evaluate these strategies are underway at our hospital.

We have previously shown serial positive mycobacterial cultures from patients on antituberculous therapy [2], [5]. In this study, 4/5 (80%) follow-up patients were positive after 2 days of treatment with antituberculous chemotherapy and between 3–6 days 5/9 (55.5%) patients were positive. However, after 7 days of chemotherapy positive cultures were rare; four samples (18.2%) were positive between 7 and 28 days of therapy and after 29 days only 2/24 (8.3%) were positive. Growth of the follow-up samples from HIV-positive patients in MODS culture was impaired, with short cords and slow growth; the clinical significance of these results is unclear. The utility of MODS for the follow-up of TBM patients on treatment, particularly as an early indicator of drug resistance requires further investigation.

The high sensitivity reported here will only be replicated if relatively large volumes of CSF are used. We have previously shown that the microbiological identification of *M. tuberculosis* in the CSF is highly volume-dependent [3]. The average volume in this study was 4.6 mls, with the deposit divided into aliquots for 4 tests. The MODS plate was inoculated with only 100 µl of CSF deposit, whereas 250 µl each was used for MGIT and LJ culture. This may account for the slightly higher sensitivity of MGIT (70.2% vs 64.9%).

**Figure 3:**
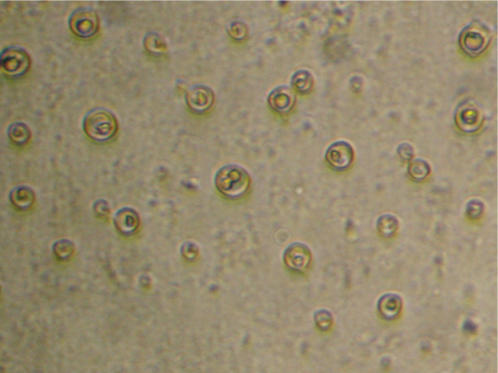
*Cryptococcus neoformans* in MODS plate at ×400 magnification with inverted microscope.

Cryptococcal meningitis is the second most common cause of meningitis among HIV-infected patients at our hospital (figure 3). Twenty-two percent (35/156) of patients here were diagnosed with fungal meningitis, all but one of whom were HIV-infected. Twenty-six of these were confirmed as cryptoccal meningitis through CSF India Ink smear or cryptococcal latex agglutination antigen test (Remel Inc., Lenexa, Kansas, USA). Dual infections with *Cryptococcus neoformans* and *M. tuberculosis* are occasionally seen in HIV–infected patients at our hospital; thus a diagnosis of cryptococcal meningitis does not exclude the possibility of TBM.

This study was performed in a tertiary referral hospital by a dedicated technician who was able to examine each plate daily for growth. In routine laboratories, optimal examination schedules would need to be established to optimise sensitivity while minimising workload. Our experience suggests a four, seven, ten and fourteen day schedule would be appropriate for CSF specimens. Moore *et al.* recently showed that rates of contamination in a high-volume laboratory are not greater using MODS than other culture techniques for the isolation *M. tuberculosis* [9], a finding supported by our study which showed only one contaminated culture in 230 samples (0.4%).

All the cultures isolated in MODS were confirmed to be *M. tuberculosis* by spoligotyping (data not shown). Isolation of atypical mycbacteria from CSF is extremely rare and optimal treatment has never been determined in clinical trials. Classical cording of *M. tuberculosis* in MODS is thought to be specific for identification in the hands of an experienced microscopist, however a rapid confirmatory test such as a nitrate strip test may be appropriate. This issue requires evaluation in a larger study.

In summary, MODS is an extremely promising technique for the rapid diagnosis of TBM, isolating *M. tuberculosis* from CSF within 10 days in the majority of cases at low cost (0.53 USD per sample). It is cheap, simple, sensitive, specific and applicable in low-technology laboratories. Its main advantage is the cheap, rapid microbiological isolation of *M. tuberculosis* from CSF and the potential to perform DST within a clinically useful timeframe.

## Materials and Methods

### Patient recruitment and sample collection

CSF samples were collected from consecutive patients (aged over 14 years) with clinically suspected TBM (defined as a combination of nuchal rigidity and CSF abnormalities) presenting to the Hospital for Tropical Diseases, Ho Chi Minh City, Vietnam between June 1st and November 1st 2006. TBM was defined as “definite” if acid-fast bacilli were seen in the CSF. It was defined as “probable” in patients with one or more of the following: suspected active pulmonary tuberculosis on chest radiography; acid-fast bacilli found in any specimen other than the cerebrospinal fluid; clinical evidence of other extra-pulmonary tuberculosis; radiological features of TBM on computed tomography (CT) scan or magnetic resonance imaging (MRI) of the brain. TBM was defined as “possible” in patients with at least four of the following: a history of tuberculosis, predominance of lymphocytes in the cerebrospinal fluid, illness duration of more than five days, a ratio of cerebrospinal fluid glucose to plasma glucose of less than 0.5, altered consciousness, yellow cerebrospinal fluid, or focal neurological signs. All patients who fulfilled the above diagnostic criteria were treated for TBM with standard antituberculous chemotherapy and adjunctive dexamethasone. All patients were tested for antibodies to human immunodeficiency virus (HIV) as part of routine care. Those who were found to be HIV-infected were screened for inclusion in an ongoing randomised double-blind placebo-controlled trial of immediate versus deferred antiretroviral therapy for the treatment of HIV-associated tuberculous meningitis. In addition to routine biochemical tests, all CSF samples were also examined by Gram-stain and India ink stain, and cultured on blood, chocolate and sabouraud dextrose agar, to exclude bacterial and cryptococcal meningitis respectively. All samples included in this study were taken as part of routine clinical care and the study did not involve any change to routine patient care and therefore consent and ethical review was not deemed necessary.

The volume of each CSF sample was recorded, the sample centrifuged at 3,000g for 15 mins, the supernatant removed and the deposit aliquoted into 4 parts. ZN smear, LJ and MGIT culture were performed on each specimen according to methods previously described [3].

### MODS

MODS testing was performed by a technician who was unaware of the clinical diagnosis, smear and other culture results. The method was performed as described in Moore *et al*. [10] with 2 minor modifications; 48 well plates were used in place of 24 and a plate sealer (Biorad optical tape, Biorad, Hercules CA, USA) was used to avoid evaporation and cross-contamination in the plate.

**Figure 4:**
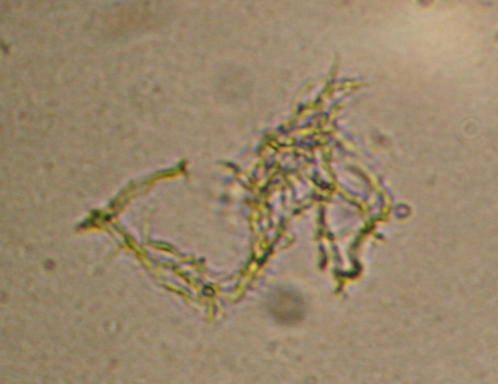
*M. tuberculosis* characteristic cording in MODS plate at ×400 magnification with inverted microscope.

MODS media was prepared with 5.9 g Middlebrook 7H9 broth (Difco, Sparks, MD), 3.1 mls glycerol and 1.25 g bacto casitone (Difco, Sparks, MD) in 880 mls sterile distilled water. The media was autoclaved and stored in 22 ml aliquots at 4°C. Each new batch was tested for sterility by incubating one aliquot at 37°C for 1 week. One MODS plate was set up each day with the addition of 2.5 mls OADC (Becton Dickinson) and 500 µl PANTA antibiotic (Becton Dickinson). Nine hundred µl media was aliquoted to each well and 100 µl CSF deposit added. One positive control (H37Rv) and one negative control well (sterile distilled water) were inoculated each day. Plates were examined daily (on weekdays) for evidence of growth (figure 4).

### Statistics

The sensitivity, specificity, positive predictive value and negative predictive value for MODS were determined and compared with ZN smear and LJ and MGIT culture, using clinical diagnosis (instigation of anti-tuberculous chemotherapy) as the gold-standard. Time to result for the three culture methods was compared using Wilcoxon signed rank test. Samples were excluded where patient medical records were not available for analysis.

### Costs

The costs of the MODS assay were calculated based upon locally purchased reagents, where possible. It was assumed 40 samples were inoculated per plate, with 1 H37Rv positive control per plate and 1 negative control per row. Labour, capital equipment costs and maintenance costs were not included in the calculations which represent only the price of consumables. An inverse microscope is approximately 2,000 USD. An exchange rate of 16,000 Vietnamese Dong (VND) was used to convert costs to US dollars.
